# The Application of Single-Cell Technologies for Vaccine Development Against Viral Infections

**DOI:** 10.3390/vaccines13070687

**Published:** 2025-06-26

**Authors:** Hong Nhi Nguyen, Isabel O. Vanderzee, Fei Wen

**Affiliations:** 1 Department of Chemical Engineering, University of Michigan, Ann Arbor, MI 48109, USA; hnhi@umich.edu; 2 Graduate Program in Immunology, University of Michigan, Ann Arbor, MI 48109, USA; ivander@umich.edu

**Keywords:** vaccine development, virus-like particles (VLPs), mRNA, recombinant protein, single-cell technology, multi-omics, infectious diseases, viral diseases, influenza, COVID-19

## Abstract

The development of vaccines against viral infections has advanced rapidly over the past century, propelled by innovations in laboratory and molecular technologies. These advances have expanded the range of vaccine platforms beyond live-attenuated and inactivated vaccines to include recombinant platforms, such as subunit proteins and virus-like particles (VLPs), and more recently, mRNA-based vaccines, while also enhancing methods for evaluating vaccine performance. Despite these innovations, a persistent challenge remains: the inherent complexity and heterogeneity of immune responses continue to impede efforts to achieve consistently effective and durable protection across diverse populations. Single-cell technologies have emerged as transformative tools for dissecting this immune heterogeneity, providing comprehensive and granular insights into cellular phenotypes, functional states, and dynamic host–pathogen interactions. In this review, we examine how single-cell epigenomic, transcriptomic, proteomic, and multi-omics approaches are being integrated across all stages of vaccine development—from infection-informed discovery to guide vaccine design, to high-resolution evaluation of efficacy, and refinement of cell lines for manufacturing. Through representative studies, we highlight how insights from these technologies contribute to the rational design of more effective vaccines and support the development of personalized vaccination strategies.

## 1. Introduction

**Figure 1:**
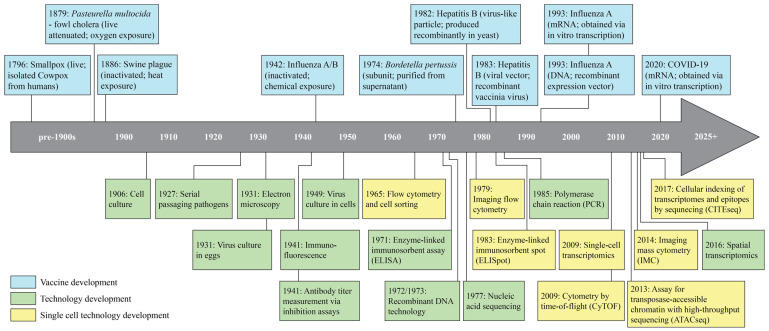
Timeline of the development of novel vaccine platforms and key laboratory techniques [3,6,7,8,9,10,11,12,13,14,15,16,17,18,19,20,21,22,23,24,25,26,27,28,29,30,31,32,33,34,35,36,37,38,39,40,41,42,43,44,45].

Vaccines have transformed healthcare and are crucial in alleviating the economic burden, with an estimated prevention of 3.5–5 million deaths per year and USD 2.9 trillion in U.S. societal savings from childhood immunization programs alone [1,2]. The availability of viral vaccines and the development of immunization programs have led to the eradication of smallpox in 1980 and the suppression and near-eradication of multiple other viruses including polio [3,4]. Edward Jenner is credited with creating the first vaccine when he observed that people infected with cowpox did not suffer from smallpox. In 1796, Jenner built on this observation by inoculating eight-year-old James Phipps with live cowpox (i.e., a live vaccine) and later challenging him with smallpox to empirically demonstrate the protection conferred by live vaccination (Figure 1) [5]. While effective, the experimental vaccination of humans with a live virus is associated with significant risk and was only possible at the time due to a lack of regulation. Over 80 years later in 1879, Louis Pasteur developed the first live attenuated vaccine after he left out a bacterial culture of *Pasteurella multocida*, resulting in attenuation via oxygen exposure [6,7]. Live attenuated vaccines are weakened versions of the original pathogen that have reduced pathogenicity but retain their immunogenicity, mitigating some of the safety concerns associated with live vaccines. A few years later in 1886, a new class of vaccines emerged: inactivated vaccines, first developed by D.E. Salmon by inactivating swine plague bacteria via heat [8]. Inactivated vaccines contain pathogens that have been killed, typically via heat or chemicals, resulting in a significantly improved safety profile relative to live or live attenuated vaccines.

These early examples of novel vaccine platform development were halting and depended primarily upon observation and empirical research rather than informed design. This changed with the advent of fundamental laboratory techniques and technologies in the early to mid-1900s including cell culture, serial passaging, and electron microscopy (Figure 1, green) [9,10,11,12,13]. Advances such as these facilitated the isolation and identification of the influenza virus in 1933, 15 years after the initial 1918 pandemic which resulted in the infection of ~500 million people and the deaths of 20–50 million [17,46]. Consequently, the first inactivated viral vaccine was developed in 1942 and approved in 1945 against influenza, supported by advances in viral culture in eggs, pathogen inactivation via formalin, and antibody titer measurements via inhibition assays (Figure 1, green) [16,17,18]. These advances also enabled the development of novel bacteria-specific vaccine platforms in the early 1900s, including toxoid and polysaccharide vaccines [47,48].

The mid-to-late 1900s was a period of significant technological advancement in the virology field across both vaccine development and evaluation. The development of immunofluorescence and flow cytometry in 1941 and 1965, respectively, unleashed for the first time single-cell analysis [49]. This provided a means to better understand the immune response to infection and vaccination and became embedded as one of the principal technologies used for analyzing immunological responses. Enzyme-linked immunosorbent assay (ELISA), developed in 1971, is now one of the primary techniques for measuring vaccine-induced correlates of protection [11,22]. Polymerase chain reaction (PCR), first developed in 1985, is still widely used across all stages of vaccine development to analyze the host response to infection and evaluate vaccine efficacy, safety, and quality [32,50].

Technological advancements during this time period also enabled the development of several new vaccine platforms beyond live, live attenuated, and inactivated vaccines. The emergence of recombinant DNA technology and nucleic acid sequencing in the 1970s led to the creation of virus-like particle (VLP) and viral vector vaccines in 1982 and 1983, respectively (Figure 1, blue) [23,24,26,27,28,29]. VLPs are similar to native viruses but lack genetic material and can be manipulated to selectively express specific proteins using genetic engineering approaches. Viral vector vaccines are produced by inserting the viral genome of a target virus into a low-pathogenic virus to display antigens of interest while preventing viral replication [51]. The utilization of recombinant DNA technology remains the predominant approach to vaccine development today. Continued advances in genetic engineering and cellular analysis informed the latest vaccine platforms—nucleic acid vaccines—which deliver viral genetic material and co-opt host machinery to produce antigens of interest. These vaccines were first developed in 1993 for influenza and first approved in 2021 for severe acute respiratory syndrome coronavirus 2 (SARS-CoV-2) [34,35,43]. These technological advances have collectively transformed and accelerated our approach to vaccine development, as demonstrated by the creation of a SARS-CoV-2 vaccine a mere one year after its emergence in 2019 compared to the nearly 25-year gap between the emergence of and vaccine development against influenza in the 1900s.

Recently, the arrival of several new single-cell technologies in the 2000s (Figure 1, yellow) has set the stage for a potential revolution in vaccine design and development. While many vaccine efforts have historically relied on empirical and bulk analyses, a detailed understanding of the immunological response to infection and vaccination is necessary for the rational design of vaccines against difficult-to-target or highly mutable viruses such as human immunodeficiency virus (HIV), hepatitis C virus (HCV), and influenza [52,53,54]. Single-cell transcriptomic, proteomic, and epigenomic technologies offer unique opportunities to analyze the immune response to viral infection and vaccination at the single-cell level and may lead to new approaches to accelerate and improve vaccine design. Although the use of single-cell technologies in the vaccinology field is in its nascent stages, many research groups are already leveraging the advantages of single-cell over bulk approaches.

In this review, we discuss select single-cell technologies, their key features, and how they have been used to enhance vaccine development, evaluation, and production. Although other single-cell technologies may be adopted for these purposes in the future, we restrict the focus of this review to technologies that have emerged as the predominant choice to date for virology and vaccinology studies.

## 2. Single-Cell Technologies

**Figure 2:**
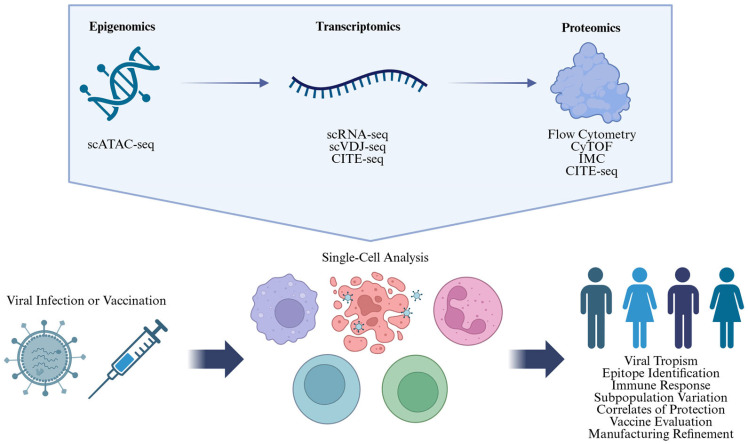
Options and applications of single-cell technologies for vaccine development. Created in BioRender. Vanderzee, I. O. (2025) https://BioRender.com/c679z0b (accessed on 14 April 2025).

Single-cell technologies enable high-resolution profiling of individual cells and span various omics modalities—including DNA (genomics), epigenetic states (epigenomics), mRNA (transcriptomics), proteins (proteomics), and metabolites (metabolomics)—each providing unique insights into cellular processes. Among these, certain omics approaches are particularly well suited for studying viral infections and vaccine responses. Upon encountering a new viral pathogen or vaccine immunogen, the immune system rapidly initiates a cascade of dynamic and tightly regulated responses. Innate immunity mobilizes within minutes to hours, followed by adaptive immunity that matures over days to weeks [55]. These responses are primarily orchestrated through non-genetic mechanisms, including epigenetic remodeling, transient transcriptional activity, metabolic shifts, effector protein expression, and post-translational modifications [56,57,58,59,60]. In contrast, genetic alterations are largely limited to lymphocyte-specific processes such as variable (V), diversity (D), and joining (J) gene segment recombination (VDJ recombination), somatic hypermutation, and class-switch recombination [61]. Given their balance of biological relevance, technical feasibility and data interpretability, single-cell epigenomics, transcriptomics, and proteomics have emerged as the most widely applied omics technologies in viral infection and vaccine research (Table 1, Figure 2).

Among these, epigenomics offers unique insights into the gene regulatory mechanisms, including chromatin accessibility, DNA methylation, and histone modifications, that determine transcriptional potential and prime cells for response to stimulation [102]. Pre-existing states of these epigenetic features influence initial viral susceptibility and vaccine responsiveness, while infection- and vaccine-induced reprogramming further shapes both immediate protection and long-term immunological memory [103,104,105]. Examples of single-cell epigenomics include single-cell bisulfite sequencing (scBS-seq) for DNA methylation, single-cell chromatin immunoprecipitation sequencing (scChIP-seq) for histone modifications, and single-cell Assay for Transposase-Accessible Chromatin sequencing (scATAC-seq) for chromatin accessibility, with scATAC-seq being the most widely used and fastest growing among them [65,106]. scATAC-seq identifies open chromatin regions by using Tn5 transposase, which simultaneously fragments accessible genomic DNA and inserts barcoded sequencing adapters [66,67]. The tagged DNA is then PCR amplified, sequenced using next-generation sequencing, and aligned to a reference genome to map regions of regulatory activity (e.g., promoters, enhancers). Single-cell resolution is achieved either through combinatorial indexing, which assigns unique barcode combinations to individual cells by redistributing them into different wells and adding a new barcode each round, or through physical isolation using microfluidics or droplet-based systems [66]. scATAC-seq generates genome-wide maps of chromatin accessibility, enabling the identification of regulatory elements that are either present in the basal state or dynamically activated or repressed in response to infection or vaccination, thereby elucidating how these epigenetic changes modulate the expression of immune-related genes across diverse cell populations [107,108,109,110].

While epigenomics reveals the regulatory landscape that shapes transcriptional potential, transcriptomics provides a direct measurement of mRNA transcript abundance and diversity [76]. In single-cell RNA sequencing (scRNA-seq), individual cells are isolated (e.g., microfluidics, sorting, plate-based), lysed, and their polyadenylated mRNA reverse-transcribed to cDNA with addition of cell-specific barcodes [76]. The cDNA is then amplified, sequenced, and mapped to quantify transcript-level gene expression. When predefined gene sets are of interest, targeted sequencing is appropriate [111,112]. Otherwise, studies investigating the immune response to infection and vaccination especially benefit from a whole-transcriptome scRNA-seq approach. By detecting transcripts from over ten thousand genes per cell [74,75,76] across thousands to hundreds of thousands of cells [72], this approach provides a global view of immune transcriptional activities (Table 1). It delineates cell subsets and infers effector states through unsupervised clustering of transcript profiles without relying on predefined surface markers or prior assumptions [76]. This data-driven approach enables the discovery of rare cell populations and unexpected regulatory pathways that may be missed in targeted single-cell analyses [76,77]. In addition, scRNA-seq enables simultaneous profiling of pathogen and host transcriptomes [70], providing insights into viral tropism and host–pathogen interactions. A specialized approach, scVDJ-seq, sequences the V, D, and J gene segments of B-cell and T-cell receptors, through targeted amplifications of their complementary-determining region 3 (CDR3) junctions. By analyzing CDR3 sequences, scVDJ-seq enables the identification and quantification of dominant clonotypes—the most expanded clonal populations defined by shared receptor sequences [81,82]. These clonotypes can serve as markers of protection or inform epitope targets for vaccine design.

An emerging advancement in transcriptomics is spatial transcriptomics, which integrates transcript-level gene expression data with spatial context, enabling precise localization of immune cell subsets and their interactions within tissue environments [113,114,115]. Spatial transcriptomics faces a trade-off between spatial resolution and transcriptome coverage. Imaging-based methods such as MERFISH use repeated cycles of fluorescently labeled probes and high-resolution microscopy to visualize and quantify spatial distribution of only targeted mRNAs at single-cell resolution [115,116]. In contrast, sequencing-based methods such as 10x Genomics Visium capture RNA transcripts from tissue sections onto spatially barcoded arrays, enabling transcriptome-wide unbiased profiling but at multi-cellular resolution (55 μm in spot diameter with 100 μm center-to-center spacing) [115,116]. Currently, no single method yet offers both single-cell resolution and unbiased transcriptome coverage without computational augmentation [114,117,118,119]. Although not yet applied to infection and vaccination studies, as spatial transcriptomics continue to mature, they will provide unprecedented insights into the spatial organization of immune responses, further enhancing our understanding of host–pathogen interactions at the tissue level.

While transcriptomics reveals abundance and diversity of RNA transcripts, understanding the functional consequences of these profiles requires proteomic analysis, as proteins, not RNA, drive cellular responses. In addition, transcript levels often do not correlate with protein abundance, and post-translational modifications are not captured by transcriptomics [120,121,122]. Single-cell proteomics encompasses technologies such as flow cytometry, Cytometry by Time-of-Flight (CyTOF), and Imaging Mass Cytometry (IMC), which quantify surface and intracellular protein expression using fluorophore- or metal-tagged antibodies (Table 1). These technologies enable the identification of immune cell subsets and effector functions through direct measurement of functional proteins such as cell-surface proteins, cytokines, or phosphorylated signaling proteins [90,92,95,99]. Additionally, infected and bystander cells can be distinguished using antibodies against viral proteins [84,123] or fluorescently labeled DNA probes targeting viral RNA [124], providing insights into viral tropism and host–pathogen interactions. Compared to epigenomic and transcriptomic approaches, single-cell proteomics offer higher throughput, enabling rapid analysis of larger cell numbers, but with lower multiplexing capacity (Table 1). Conventional flow cytometry, in use since the 1970s (Figure 1), relies on bandpass filters to detect narrow portions of fluorophore emission spectra, typically limiting panels to 5–10 markers due to spectral overlap [89,125]. In rare cases, carefully optimized panels can reach up to 28 markers [88]. Spectral flow cytometry overcomes this limitation by capturing full emission spectra and computationally unmixing overlapping signals, allowing detection of 20–30 markers, with some optimized panels reaching up to 40 markers [91,92]. From here on, “flow cytometry” refers specifically to the conventional method, and we include only studies using panels with more than 10 markers. CyTOF further expands multiplexing capacity, routinely detecting over 40 markers, while IMC adds valuable spatial context (Table 1). However, these antibody-dependent methods are restricted to pre-selected protein panels, prioritizing specificity over comprehensiveness, inherently biasing findings toward well-characterized targets [126].

Emerging single-cell mass spectrometry (scMS) technologies aim to bridge this gap by enabling unbiased, comprehensive proteome profiling [127,128,129,130]. In a typical scMS workflow, individual cells are isolated and lysed in nanoliter-scale reactors, peptides are prepared by enzymatic digestion and then identified by liquid chromatography coupled with ultra-sensitive mass spectrometry [127]. Current scMS methods can quantify approximately 1000–3000 proteins per cell [130,131]. However, they remain largely inaccessible due to high cost and technical complexities. Challenges include low protein quantities per cell, high risk of sample loss, limited sensitivity of instruments, and difficulties in data analysis [127,131,132,133]. More details on their development and current limitations can be found in recent reviews [132,133].

Compared to other omics approaches, genomics and metabolomics are less commonly used in infection and vaccine studies. Single-cell DNA sequencing (scDNA-seq) is primarily applied in fields where resolving cellular genomic heterogeneity is critical, such as cancer biology and organismal and developmental biology [134]. Its limited use in infection and vaccination studies stems from its focus on static genomic information, which may not capture the dynamic processes involved in immune responses. Nevertheless, future applications could include studying DNA viruses, such as HPV and herpesviruses, or retroviruses like HIV, where it may help characterize proviral genome heterogeneity, viral integration sites, and virus-induced mutations [135]. Another potential application is to identify the host genetic factors that influence susceptibility to infection or responses to vaccines. On the other hand, while single-cell metabolomics, primarily reliant on mass spectrometry, holds great promise for uncovering metabolic shifts that influence immune cell activation, differentiation, and effector functions [136], its adoption has been greatly hindered by technical barriers. These include low metabolite abundance, rapid turnover rates, and limited computational tools [137,138]. Addressing these challenges will be essential for enabling the broader application of single-cell metabolomics in infection and vaccine research.

Each omics approach provides distinct yet complementary insights into immune responses. Integrating multiple modalities, such as combining transcriptomic and proteomic data, enables a more comprehensive, multi-layered understanding of immune responses [139,140]. One prominent single-cell multi-omics technology is Cellular Indexing of Transcriptomes and Epitopes by Sequencing (CITE-seq), which combines detection of hundreds of surface proteins with unbiased transcriptome profiling in thousands of single cells [42] (Table 1). Cells are first stained with antibodies against specific surface proteins [42,139,140]. Each antibody is conjugated to a unique DNA oligonucleotide known as an antibody-derived tag (ADT). Individual cells are then isolated into microfluidic droplets or microwells containing beads coated with uniquely barcoded oligonucleotides. Upon cell lysis, released ADTs and polyadenylated mRNA are captured by these barcoded oligos, reverse-transcribed into cDNA, and sequenced. Integrating transcriptomic and surface protein data from the same cell enables CITE-seq to resolve cellular phenotypes with greater precision, particularly for closely related subsets that may be indistinguishable by either modality alone [42,139,140]. While CITE-seq is still limited by specific antibody availability, its use of DNA-barcoded antibodies allows for significantly greater multiplexing than spectral flow cytometry or CyTOF, which are restricted by fluorophores and metal tags, respectively (Table 1) [42,92,96]. Other multi-omics approaches exist, as reviewed elsewhere [139,140,141], but we focus on CITE-seq for its broad applications in infection and vaccination studies.

## 3. Harnessing Single-Cell Technologies to Advance Vaccine Development

**Figure 3:**
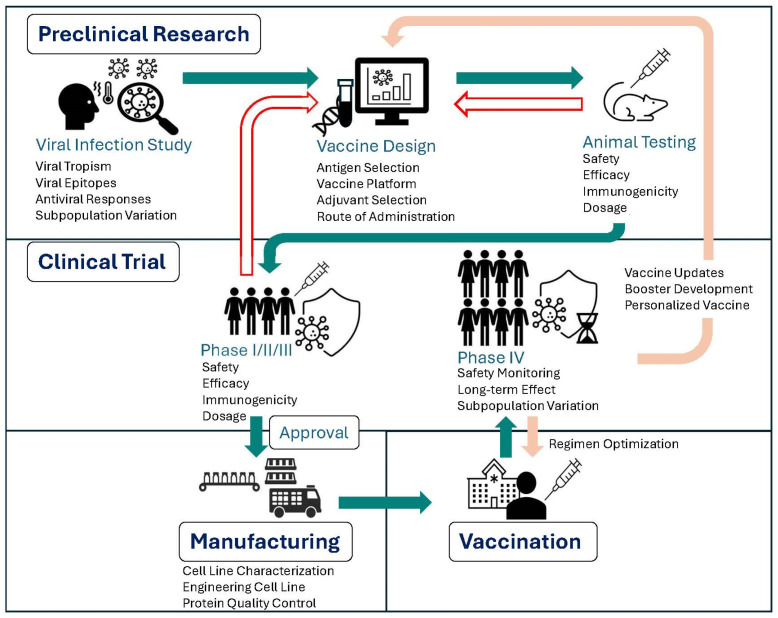
Stages of vaccine development. Green arrows indicate standard sequential progression. Red arrows indicate failure-driven return to earlier stages. Orange arrows indicate information flow that refines prior stages.

Single-cell technologies are transforming how researchers study immune responses to viral infections, offering unprecedented resolution into cellular heterogeneity. These technologies are now increasingly being applied across the vaccine development pipeline, ranging from uncovering mechanisms of immune activation to evaluating vaccine efficacy and optimizing manufacturing processes (Figure 3). Below, we highlight representative studies that demonstrate how single-cell technologies are shaping the field of vaccinology, accelerating both discovery and application. Specifically, we cover how single-cell technologies have been utilized to enhance vaccine development, evaluation, and manufacturing.

### 3.1. Informing Vaccine Development via Analyses of Immune Response to Viral Infection

#### 3.1.1. Determining Viral Tropism

Understanding viral tropism—the specific cell types a virus infects—can guide targeted vaccine design by optimizing antigen delivery strategies. For example, a previous study leveraged knowledge of viral tropism to develop a polio:rhinovirus chimera viral vector vaccine that exhibits tropism for dendritic cells (DCs), thus improving DC activation, antigen presentation, and priming of T cell immunity [142,143]. Separate groups have modified vesicular stomatitis virus (VSV)-based viral vector vaccines to limit their neurotropism and consequently improve their safety profiles, resulting in a relatively safe VSV-based Ebola virus vaccine and a VSV-based Nipah virus vaccine [144,145]. Traditionally, viral tropism has been assessed using techniques such as microscopy, immunohistochemistry, flow cytometry, PCR, and single-genome sequencing [146]. However, these methods rely on bulk analyses or have limited multiplexing capacity, which prevents the comprehensive and unbiased identification of cell subsets targeted by viruses. Single-cell technologies are capable of highly multiplexed analyses with simultaneous evaluation of many tissues, cell types, and viral markers, which can reveal new insights into viral tropism and unlock novel approaches for vaccine design.

Single-cell proteomic approaches are often used for viral tropism studies as they are high-throughput and offer relatively reliable cell phenotyping by measuring proteins: direct mediators of biological activity (Table 1). As an example, spectral flow cytometry of peritoneal cells from mice infected with a reporter strain of murine gammaherpesvirus 68 (MHV68) led to cell type identification using canonical protein markers and revealed dominant MHV68 infection of large peritoneal macrophages, along with infection of small peritoneal macrophages, DCs, and B cells [147]. Parallel to single-cell suspension proteomic technologies like flow cytometry, single-cell imaging proteomic technologies such as IMC allow for the direct analysis of minimally processed tissue sections and are well-suited for analyzing limited samples such as human tissue biopsies. For example, IMC analysis of liver biopsies from hepatitis B virus (HBV)-infected patients confirmed preferential infection of hepatocytes based on the presence of hepatitis B surface antigen (HBsAg) and hepatitis B core antigen (HBcAg), demonstrating the applicability of IMC for viral tropism studies [148]. In a separate study using an IMC panel that included antibodies for cell phenotypic protein markers and the SARS-CoV-2 spike protein (S-protein), IMC analysis of lung tissue from COVID-19 patients confirmed preferential infection of alveolar epithelial cells [149]. Beyond the primary target of epithelial cells, several other cell types were S-protein positive, including macrophages, smooth muscle cells, and neutrophils, indicating that SARS-CoV-2 exhibits tropism for multiple cell types within the lung.

Among highly multiplexed single-cell technologies, scRNA-seq is most commonly employed to study viral tropism due to its higher multiplexing capacity relative to proteomic approaches. This supports an unbiased analysis that does not require pre-selecting markers and allows for simultaneous sequencing of both viral and host transcriptomes, enabling the identification of virus-infected cells. However, further validation via proteomic analysis is important, as gene expression heterogeneity and variable correlation between transcript and protein levels can confound transcript-based phenotyping [150,151]. As an example, scRNA-seq was used to determine infected cell types from influenza A virus (IAV)-infected mouse lung by manually annotating cell clusters based on expression of canonical mRNA cell markers (e.g., *Sftpc* for epithelial cells, *Col1a1/Col1a2* for fibroblasts, *Cd3e/Cd3d* for T cells, etc.) and simultaneously detecting IAV mRNA [152]. This analysis demonstrated that IAV exhibits tropism for multiple cell types, including endothelial cells, granulocytes, and B cells, in addition to its well-established preference for epithelial cells. Flow cytometry analysis using antibodies against the intracellular IAV nucleoprotein (NP) confirmed these infected cell populations from a proteomic approach. Similarly, scRNA-seq analysis of host and viral transcriptomes of Japanese encephalitis virus-, human BK polyomavirus-, and infectious bronchitis virus-infected tissue revealed different tropism for neurons, proximal tubule cells, and distal tubules/collecting duct cells, respectively [153,154,155]. In addition to determining viral tropism by directly detecting infected cells through the presence of viral mRNA, potential target cell types can also be identified by assessing the mRNA expression of viral entry receptors. As an example, an analysis of human scRNA-seq data revealed several tissues contain cell types that express *ACE2* and *TMPRSS2*, the entry receptor and S-protein primer for SARS-CoV-2, respectively, including hepatocytes, endodermal cells, enterocytes, and goblet cells [156]. This is consistent with clinical manifestations of COVID-19, including gastrointestinal symptoms and kidney failure. These findings support vaccine approaches that promote systemic immunity, such as subcutaneous or intramuscular administration, over approaches targeting the respiratory tract, such as inhaled or intranasal vaccines [157].

#### 3.1.2. Identifying Viral Epitopes

During viral infection, B and T cells recognize viruses and infected cells by binding to specific regions of viral antigens called epitopes. This epitope binding, along with coreceptor and cytokine receptor binding, initiates B and T cell activation and is essential for mounting an antiviral adaptive immune response. Consequently, identifying immunogenic viral epitopes is critical for the rational design of vaccines that can robustly activate the adaptive immune system. Given that most viral epitopes are peptide-based, with B cells recognizing unprocessed, conformational peptides on the viral surface and T cells recognizing processed, linear peptides presented on Major Histocompatibility Complexes (MHCs) on antigen-presenting cells (APCs), there is a growing interest in recombinant peptide vaccines [158]. Recombinant vaccines offer several advantages over live attenuated or inactivated vaccines, including customizable peptide design (e.g., selective inclusion/exclusion of epitopes, multi-epitope constructs), improved safety, easier storage, and highly scalable, cost-effective production methods [159,160]. As a result, many epitope-based vaccines have been developed using insights gained from epitope mapping techniques [161,162,163]. This epitope-based approach is especially promising for highly mutable viruses such as HIV, IAV, and HCV, which may require vaccines that target highly conserved epitopes and elicit broadly neutralizing antibodies [164,165,166].

Several experimental approaches have traditionally been used to identify immunogenic viral epitopes. Structural analysis methods such as X-ray crystallography, nuclear magnetic resonance, cryo-electron microscopy, and mass spectrometry can provide detailed epitope mapping, with resolution of an antigen–antibody complex at up to 1.2 Å, but are generally low-throughput (e.g., single or several epitopes), costly, and can take several months [167,168]. For B cells, higher-throughput approaches include deep mutational scanning (DMS), peptide microarrays, bacteriophage peptide display technology, and bacterial or yeast surface display technology. T cell epitopes, on the other hand, have been identified using peptide–MHC multimers, microsphere-assisted peptide screening, cell-based selection platforms, and cell surface display technology [169,170,171]. These approaches generally target a single antigenic protein at a time and can require the isolation and expansion of B or T cell clones to identify epitopes, making the process costly and time-consuming (weeks to months) [168,172,173,174]. Recently, scRNA-seq approaches have emerged as a powerful tool to simultaneously analyze hundreds of virus-specific B and T cell populations and their corresponding epitopes [79,175]. ScVDJ-seq in particular is critical for epitope identification, as the VDJ gene segments encode the epitope-binding regions of BCRs and TCRs, allowing for the identification of clonotypes based on shared VDJ sequences. Given that scVDJ-seq does not require cell isolation or expansion and can be applied to heterogeneous cell populations, it can screen all clonotypes simultaneously, supporting an analysis of hundreds of viral epitopes with significantly higher comprehensiveness relative to traditional approaches.

As an example, scTCR-seq analyses of human peripheral blood mononuclear cells (PBMCs) identified over 900 significantly enriched T cell clonotype groups in COVID-19 patients [79]. Computational pairing of these TCR clones with peptide–MHC complexes spanning 866 9-mer peptides from 11 SARS-CoV-2 proteins revealed over 1600 TCR–peptide pairs that were significantly shared by COVID-19 patients, totaling 31 CDR3 groups and 114 peptides. Notably, more than 90% of the 114 enriched peptides were located within the open reading frame 1a, 1b, and 3a proteins (ORF1ab, ORF3a), the S-protein, and the nucleocapsid protein (N-protein), indicating immunodominance of these proteins. A similar approach for B cells is possible, as computational mapping approaches have been developed to predict B cell epitopes based on BCR sequences [176]. Although these results are based on computational predictions and require further experimental validation, this study demonstrates the potential of in silico epitope mapping to VDJ sequences at the single-cell level to identify candidate antigenic epitopes for vaccine development.

Beyond computational mapping, a specialized technique called Linking B cell Receptor to Antigen specificity through sequencing (LIBRA-seq) simultaneously maps BCR sequences and their corresponding antigens at the single-cell level [177]. To achieve this, antigens of interest are conjugated to a unique DNA barcode and are incubated with B cells. B cells specific for the barcoded antigens will bind to them, and both their BCRs and antigen-associated DNA barcode are sequenced, allowing for BCR matching to specific antigens. To expand on this method to identify specific B cell epitopes within an antigen, epitope-specific variants can be incorporated into the antigen panel. As an example, LIBRA-seq was applied to PBMCs from HIV-1-infected donors using a panel of DNA-barcoded HIV-1 envelope glycoprotein (Env) epitope-specific variants [175]. This approach identified several BCRs targeting CD4bs and V3-glycan epitopes of Env, which were further validated with ELISA. This proteomic validation of antigen specificity is critical, as the presence of a barcode in the sequencing data does not guarantee true, high-affinity BCR binding to the antigen [178]. Notably, LIBRA-seq analysis predicted conformational epitope specificity for either the trimeric Env protein, the monomeric protein, or both, which was also validated via ELISA. These insights distinguish LIBRA-seq from most other epitope mapping methods, which typically assess linear but not conformational epitopes [167]. While still in its nascent stages, the use of high-throughput, comprehensive single-cell transcriptomic technologies for immunogenic viral epitope discovery can unlock new strategies for epitope-based vaccine design.

#### 3.1.3. Comparative Analyses of Responders vs. Non-Responders

Different viruses elicit distinct immune profiles, broadly defined by Type 1, Type 2, or Type 3 immunity and the production of specific antibody isotypes (e.g., IgG, IgA) [179,180]. The immune response elicited by a vaccine can be skewed towards these different profiles based on the selection of adjuvants, route of administration (RoA), and vaccine platform. For example, adjuvants that bind to specific pattern recognition receptors (PRRs) on APCs can bias the immune response towards Th1, Th2, or Th17; enhance cross-presentation; and promote cytotoxic T cell (CTL) production [181]. When selecting a route of administration, intramuscular and subcutaneous routes typically induce systemic immune responses and IgG production, while mucosal immunization (e.g., oral, intranasal, intrapulmonary) induces localized protection with IgA production [157]. Vaccine platform choice further influences the immune response, as different platforms elicit varying profiles, magnitudes, and durations of both cellular and humoral immunity [182]. A significant challenge in rational vaccine design that leverages these tunable vaccine components is the limited understanding of disease-specific mechanisms underlying protective immunity [183]. Comparative analyses of immune responses in individuals with robust versus poor viral control can reveal key signatures of protective immunity. Although the direct application of insights from comparative analyses to vaccine design has been limited thus far, these approaches hold significant promise for advancing vaccine development as our toolkit and understanding of disease-specific immune mechanisms continue to expand and evolve [184]. Given the high degree of immune system heterogeneity, single-cell technologies are especially powerful for dissecting these variable responses and identifying the cellular and molecular features that distinguish effective immunity.

As previously discussed, proteomic single-cell technologies are reliable for cell phenotyping and provide direct insights into cell states by analyzing proteins. As an example, spectral flow cytometry analysis of human blood and bronchoalveolar lavage fluid (BALF) from IAV- and COVID-19-infected patients enabled clear immune cell phenotyping based on canonical protein expression [185]. This revealed dysregulated inflammatory immune responses in the lower airways of severe patients vs. non-severe patients, characterized by increased infiltration of CD14^low^CD16^+^ nonclassical monocytes, activated CD4^+^ and CD8^+^ T cells, and plasmablast B cells. Further investigation using a human cytokine panel revealed elevated inflammatory cytokines and chemokines in the BALF of severe COVID-19 and IAV patients, including IL-6, IL-8, MCP-1, MIG, IP-10, IL-12 and MIP-1β, which is indicative of an unresolved pro-inflammatory Th1 immune response. In patients with moderate disease, these cellular and cytokine alterations were absent, likely reflecting more effective viral control and a rapid resolution of the inflammatory response by the time of sample collection. Overall, these findings indicate that sustained Th1-skewed inflammation is associated with severe manifestations of viral respiratory infections, whereas timely resolution of inflammation is critical for favorable clinical outcomes. Several studies have shown that adjuvant-based strategies, such as those employing TLR4 and TLR9 agonists (e.g., CpG ODN 1018, AS01, and AS04), can enhance Th1-biased immunity via activation of APCs and consequently support the rapid clearance of viruses upon infection and timely resolution of inflammation. Based on the findings of this study, such approaches are promising for improving the efficacy of vaccines against IAV and SARS-CoV-2.

In a separate study, scRNA-seq analysis of the BALF from severe and mild COVID-19 patients revealed distinct immune cell signatures between the two groups, including significant enrichment of plasmacytoid dendritic cells (pDCs), alveolar macrophages (AMs), and CD8^+^ T_rm_ cells in mild patients, while neutrophils, FCN1^+^ monocytes, monocyte-derived SPP1^+^ macrophages, NK cells, and naïve CD4^+^ T cells were significantly enriched in severe patients [186]. Further characterization of these cell populations revealed enriched mRNA expression associated with the naïve CD4^+^ T cells in severe patients (e.g., *IL7R*, *CCR7*, *S1PR1*, and *LTB*) and the CD8^+^ T_rm_ cells in mild patients (e.g., effector molecules *XCL1*, *ITGAE*, *CXCR6*, and *ZNF683*). Additionally, the severe-enriched monocyte-derived SPP1^+^ macrophages had upregulated mRNA expression of inflammatory chemokines (*CCL2*, *CCL3*, *CCL4*, *CCL7*, and *CCL8*) and hypoxia and oxidative stress markers (*HMOX1* and *HIF1A*) and downregulated expression of MHC II (*HLA-A* and *HLA-DQA1*) and type I IFN mRNA (*IFIT1* and *OAS1*). Together, this analysis suggests that severe COVID-19 is associated with impaired APC activation, reduced antigen presentation, and suboptimal T cell priming and expansion. These findings underscore the potential utility of adjuvants, such as those described in the previous study, to enhance APC function and T cell activation, thereby improving immune control of SARS-CoV-2 infection.

Given that single-cell proteomic and transcriptomic approaches each offer unique advantages, multi-omic strategies can provide a more comprehensive understanding of complex immune responses by integrating multiple layers of information such as chromatin accessibility, RNA expression, and protein abundance. As an example, clustering based on canonical protein and mRNA markers from a CITE-seq analysis of PBMCs from COVID-19 patients led to the identification of 18 distinct cell types using manual annotation, as well as several sub-clusters within each cell type [100]. Differences in cell composition significantly correlated with disease severity, with expansions in proliferative CD4^+^ and CD8^+^ T cells and reduction in γδ T cells associated with severe disease. Additionally, Th1 cells were enriched in asymptomatic patients, while CD8^+^ effector T cells were enriched in severe patients, suggestive of poorly controlled inflammation. Several proteins were associated with severe/critical patients, including CCL4, CXCL10, IL-7 and IL-1. Despite an increase in plasma cells, scBCR-seq data revealed that IgA2 was significantly reduced in symptomatic patients relative to asymptomatic patients, indicative of insufficient humoral response. The enrichment of Th1 cells and IgA2 in asymptomatic patients further supports the potential benefit of Th1-skewing adjuvants, as previously discussed, as well as the use of mucosal vaccination strategies, which have been shown to elicit stronger IgA responses than non-mucosal routes of administration [157].

While the direct translation of findings from these comparative analyses into vaccine design is still emerging, these studies highlight the ability of single-cell technologies to uncover nuanced differences in immune responses between patients with severe and moderate disease. Such insights can help identify the specific immune features that vaccines should target to achieve optimal viral control.

#### 3.1.4. Analyzing Subpopulation Variation

The immune response to viral infection and vaccination can vary significantly across subpopulations based on differences in intrinsic host factors such as age, sex, comorbidities, and genetics, as well as extrinsic, behavioral, nutritional, and environmental factors [187]. As a result, there is growing interest in developing personalized vaccines that account for an individual’s unique background and immunological profile to optimize vaccine safety and efficacy [188]. Although most personalized vaccines in clinical development today are being developed for cancer immunotherapy [189], the development of personalized antiviral vaccines is an active area of investigation. The breadth and granularity that single-cell analyses offer make them well-suited for examining immune responses to natural infection across subpopulations to reveal variations that can be targeted for personalized vaccine development.

As an example, a CyTOF analysis of influenza-infected mice using a 40-marker panel designed to identify 25 canonical immune cell types and their functional states revealed significant differences in the antiviral response based on age and genetic background [84]. Evaluation of the immune response to IAV across mouse strains with distinct genetic backgrounds revealed that BALB/c mice exhibited enhanced survival compared to C57BL/6 mice. This enhanced protection was associated with increased frequencies of γδ T cells, eosinophils, and CD317^+^ and IL-10^+^ immune cells. At 3 days post-infection, BALB/c mice had elevated levels of inflammatory cytokines IFNγ and GZMB and elevated levels of the anti-inflammatory cytokine IL-10 at 6 days post-infection. These findings suggest that BALB/c mice exhibit more effective regulation of the antiviral response, characterized by an early, robust Th1-skewed inflammatory phase followed by timely suppression mediated by IL-10, reflecting a negative feedback mechanism previously described in the context of antiviral immunity [190]. Comparison of aged and young C57BL/6 mice revealed pronounced deficiencies in T and NKT cell populations in aged mice, indicating an age-related decline in cellular immunity that likely contributes to reduced survival. These findings collectively suggest that vaccines designed to elicit strong Th1 responses may improve IAV infection outcomes, particularly in genetically susceptible populations, while strategies aimed at enhancing T cell immunity are critical for protecting older individuals. From a transcriptomic approach, a scRNA-seq analysis of BALF and PBMC samples from both obese and nonobese COVID-19 patients revealed a reduced inflammatory response in obese individuals based on decreased expression of inflammatory mRNA markers associated with IFNα, IFNγ, and TNFα responses, as well as lower levels of immune-recruiting chemokine mRNA markers such as CXCL10 [191], which are associated with a Th1 response [190]. Indeed, other studies have revealed that the cellular response of obese individuals is defective and skewed towards a Treg and Th2 phenotype rather than Th1 [192,193], which is critical for antiviral immunity. These findings suggest that vaccine designs that elicit a robust Th1 response may be beneficial for obese individuals.

### 3.2. Evaluating Vaccine Efficacy and Safety via Immunogenicity Analysis

After identifying vaccine candidates, the next step is to evaluate their efficacy and safety, starting with animal testing and progressing to clinical trials (Figure 3). Although regulatory approval of vaccines ultimately depends on demonstrating clinical efficacy and safety in randomized controlled trials—such as reducing symptomatic infections, severe diseases, viral shedding, and absence of adverse events [194]—immunogenicity studies play an essential complementary role. By measuring the immune response a vaccine generates, including innate, humoral and T cell responses, immunogenicity data reveal whether a vaccine engages appropriate immune pathways and effector functions required for protection [195,196]. Single-cell technologies have enhanced immunogenicity assessment by enabling high-resolution profiling of immune responses, uncovering functional heterogeneity, rare subsets, and clonal dynamics that are often masked in bulk analyses. In the following sections, we review how single-cell analysis of immunogenicity data has advanced the discovery of potential correlates of protection (CoPs), enabled detailed characterization of responses to specific vaccine components and mechanisms underlying adverse events, and provided insights into the immune responses of immunocompromised populations.

#### 3.2.1. Discovering CoPs

CoPs—immune features that reliably predict vaccine-induced protection—are essential for evaluating and advancing vaccines. Establishing CoPs helps de-risk vaccine development by providing early indicators of efficacy before large-scale trials [197]. CoPs are particularly valuable for pathogens with low incidence, sporadic outbreaks, or ethical barriers to human challenge studies, such as Nipah virus [197]. Currently, neutralizing antibody titers remain the only widely accepted and regulatory-approved CoPs, serving as surrogate endpoints for several viral vaccines, such as those against influenza, measles, rabies, polio, hepatitis A and B [198,199,200,201,202,203]. Surrogate endpoints enable clinical immunobridging, allowing efficacy to be inferred across products or populations without the need for full-scale trials [197]. However, antibody titers alone overlook contributions from the cellular components of immune responses, are not applicable to all pathogens, and are not always reliable predictors of viral control across diverse serotypes or patient populations, highlighting the need for more robust alternative markers [198,204]. With granular insights into cellular responses, single-cell technologies have expanded the range of immune parameters to be explored as CoPs, such as protective cell subsets and transcriptional signatures.

To address the limited predictive value of antibody titers, a CyTOF-based analysis of PBMCs from a Phase II trial compared immune cell subset composition induced by non-traditional oral influenza vaccine (VXA-A1.1) to that induced by a standard injected inactivated vaccine (IIV) or placebo, followed by viral challenge [205]. For VXA-A1.1, protection from viral shedding was primarily associated with the day 8 post-vaccination frequencies of total plasmablasts and specific plasmablast subsets, including integrin α4β7^+^, CD62L^low^, pSTAT5^+^, and HA^+^ plasmablasts. In contrast, for IIV, protection correlated with traditional antibody measures, such as HAI and microneutralization titers. Interestingly, although IIV induced more total and HA^+^ plasmablasts than VXA-A1.1, these did not correlate with protection for IIV recipients. These findings demonstrate that immune cell subsets are more appropriate CoPs for oral influenza vaccines, while antibody responses serve as reliable CoPs for injected vaccines.

Another CyTOF-based study tracked B and T cell responses of ten individuals following two doses of SARS-CoV-2 BNT162b2 vaccine [83]. After the second dose, effector memory CD4^+^ and CD8^+^ T cells exhibited the greatest expansion, characterized by high expression of activation markers ICOS and CD38, the proliferation marker Ki67, and multiple markers of metabolic transporters, while naïve T cells contracted. Similarly, B cell subsets—including activated IgM^+^ B cells, memory B cells, and plasmablasts—expanded, while naïve B cells contracted. In addition, Pearson correlations revealed significant associations between the ICOS^+^CD38^+^CD8^+^ and CD4^+^ T cells with plasmablast frequencies, demonstrating a coordinated adaptive immune response. Interestingly, only ICOS^+^CD38^+^ CD8^+^ T cells significantly correlated with SARS-CoV-2 IgG titers, which correlated with neutralization titer, while plasmablasts trended toward significance and no correlation was observed for CD4^+^ T cells. Although not directly involved in antibody production, the correlation of ICOS^+^CD38^+^CD8^+^ T cell frequency with IgG titers indicates they serve as a marker of a coordinated and robust protective immune response involving both cellular and humoral components. Notably, one participant who later experienced breakthrough infection had the lowest abundance of ICOS^+^CD38^+^CD8^+^ T cells, further supporting its potential as a CoP. However, this observation requires validation in larger, more diverse cohorts before broader conclusions can be drawn.

In addition to immune cell subsets, transcriptional signatures have also been explored as potential CoPs, offering insights into the functional pathways underlying effective vaccine responses. For example, scTCR-seq analysis of vaccine-induced CD38^+^HLA-DR^+^CD8^+^ T cells from a Phase I trial of TAK-003 dengue vaccine revealed that a large percentage of dominant memory clones detected at day 120 post-vaccination were already present at day 14, identifying these as memory precursors [69]. scRNA-seq further revealed that these memory precursors were clustered within a distinct transcriptional group enriched for cytotoxic effector programs (such as *GZMA*, *GZMB*, and *PRF1*) and cellular metabolism (such as *TYMS*, *IDH2*, and *GAPDH*), all of which are regulated by the mTOR signaling pathway. Guided by this metabolic signature, the study further identified *TFRC*, the transcript encoding iron transporter TfR1/CD71, which is upregulated by mTOR, as a robust marker highly enriched on CD8^+^ T cells destined to become memory cells. Flow cytometry analysis confirmed TfR1 expression on CD8^+^ T cells correlated with higher intracellular levels of proteins associated with proliferation, cytolytic function, and memory lineage commitment. Although the study did not establish whether upregulation of *TFRC* or metabolic transcripts is directly associated with protection against dengue infection, these transcripts were correlated with the induction of effector memory CD8^+^ T cells. Other viral infection studies have demonstrated that effector memory CD8^+^ T cells contribute to protection by reducing disease severity and improving viral control [206,207,208,209,210], and vaccine studies further highlight the importance of effector memory CD8^+^ T cells in enhancing protection [211,212]. Together, these findings suggest that *TFRC* and related metabolic transcripts are promising CoPs and support further validation of their predictive value.

#### 3.2.2. Characterizing Immunogenicity of Vaccine Components

As discussed in Section 3.1, informed by insights from natural infection, vaccines can be rationally designed to effectively engage the immune system by optimizing components such as epitopes, adjuvants, platforms and RoA. To move toward more targeted and effective vaccine strategies, it is critical to evaluate in detail how each of these components shapes the immune response. In this section, we highlight how single-cell technologies have been applied to profile immune responses to specific components, specifically adjuvants, platforms, and RoA, offering high-resolution insights that inform future vaccine development.

Many inactivated vaccines and those based on recombinant antigens rely on coadministration with an adjuvant to elicit a robust immune response [181,213]. Adjuvants enhance innate immune activation, which in turn amplifies the magnitude and quality of adaptive immunity [213]. Although adjuvants are essential for the efficacy of many vaccines, identifying the most effective adjuvant–immunogen combinations remains an ongoing challenge, prompting comparative single-cell studies assessing how different adjuvants, or the presence versus absence of an adjuvant, shape immune responses to a given immunogen. For example, spectral flow cytometry was used to compare the immune responses in mice immunized with SARS-CoV-2 spike-ferritin nanoparticle (SpFN) vaccine formulated with either Aluminum Hydroxide (AH) or Army Liposome Formulation containing QS-21 (ALFQ) as adjuvant [214]. This approach discriminated twelve innate immune subsets and revealed markedly increased frequencies and activation of APCs in the ALFQ group, including conventional DC subsets (cDC1 and cDC2), pDCs, monocytes, and monocyte-derived DCs. Enhanced expression of costimulatory markers (CD80, CD86, CD40) further indicated robust functional activation of classical APCs and macrophage subsets. In addition, ALFQ promoted higher frequencies of effector memory CD4^+^ and CD8^+^ T cells. Notably, ALFQ induced significantly higher percentages of polyfunctional spike-specific CD8^+^ T cells co-expressing IFNγ, IL-2, and TNFα, whereas AH primarily elicited single cytokine-secreting T cells. These findings support ALFQ as a potent adjuvant for SpFN by enhancing both the magnitude and quality of innate and adaptive responses.

Another study employed CyTOF, scATAC-seq and scRNA-seq to characterize the epigenomic changes 42 days after immunization in a Phase II trial of an AS03-adjuvanted versus unadjuvanted inactivated H5N1 influenza vaccine [215]. CyTOF analysis targeting histone modification proteins revealed that vaccination with H5N1 + AS03, but not H5N1 alone, induced a significant reduction in several histone acetylation marks (H3K27ac, H4K5ac, and H3K9ac) and PADI4, an arginine deiminase involved in histone citrullination, in classical monocytes. These epigenetic changes were associated with diminished production of innate cytokines such as TNF-α, IL-1β, IL-12, and IL-10, signifying innate immune refractoriness. Consistent with this, scATAC-seq analysis showed that the AS03-adjuvanted group had reduced chromatin accessibility at loci targeted by AP-1 transcription factors, key regulators of early innate inflammatory responses. Notably, this was accompanied by increased accessibility at loci targeted by IRF and STAT families of transcription factors, which are associated with expression of IFN- and antiviral-related genes. Supporting these findings, scRNA-seq revealed reduced expression of AP-1 family members (*FOS*, *JUN*) and elevated expression of *IRF1* and *STAT1*. These results suggest that the addition of AS03 to the H5N1 vaccine modulates the innate epigenetic landscape to suppress excessive inflammation while enhancing antiviral vigilance, potentially minimizing host damage during the later stages of infection and contributing to more effective vaccine-induced protection.

In contrast to vaccines based on protein or inactivated virus, mRNA vaccines constitute a self-adjuvanted platform in which both the mRNA and its lipid nanoparticle (LNP) delivery system actively engage innate immune pathways to enhance immunogenicity [216,217,218]. scRNA-seq analysis of injection site tissues from mice collected 16 h after intramuscular administration of LNP-formulated mRNA vaccine or LNP co-delivered with the matched spike protein subunits revealed distinct innate immune signatures [216]. Both platforms activated stromal cells, leading to upregulation of pro-inflammatory cytokine transcripts such as *Il6*, *Tnf*, and *Ccl2*—a response attributable to the LNP component, as confirmed by empty LNP control. Despite delivering the same antigen, only the LNP + mRNA platform induced robust type I IFN responses, characterized by increased expression of type I IFN-responsive gene transcripts (*Isg15*, *Oasl1*, *Ifit3*) in migratory DCs. This response was linked to localized expression of *Ifnb1* transcripts, detected exclusively in spike-mRNA^+^ fibroblasts, identifying mRNA as the trigger of IFNβ induction and downstream type I IFN responses. These findings highlight that the molecular and functional features of a vaccine platform influence innate immune activation, underscoring the importance of platform selection in shaping early immune responses.

Besides adjuvants and platforms, RoAs are also known to affect the quality of vaccine responses [219,220,221]. CyTOF analysis of PBMCs from macaques vaccinated with Modified Vaccinia virus Ankara (MVA) via subcutaneous (SC), intramuscular (IM), or intradermal (ID) routes revealed route-specific T cell profiles [222]. IM vaccination induced the strongest polyfunctional CD8^+^ T cell responses, characterized by expression of IFNγ, IL-10, MIP1-β, TNFα, and IL-2. It also elicited robust Th1-like CD4^+^ T cell responses, marked by high levels of CD154, CD69, IFNγ, TNFα, and IL-2. ID vaccination produced intermediate responses, with enrichment of cytotoxic (PRF1^+^, GZMB^+^) and migratory CD8^+^ T cell clusters, as well as cytotoxic CD4^+^ subsets. SC vaccination elicited minimal CD8^+^ T cell responses and lower frequencies of polyfunctional CD4^+^ T cells. These results demonstrate that RoAs have distinct effects on adaptive immunity and should be selected carefully to elicit desired responses.

Together, these comparative studies demonstrate how single-cell technologies can identify immunological signatures associated with specific adjuvants, platforms, and RoAs. As such datasets accumulate, they will help establish a framework to guide vaccine design by enabling the optimal selection of components to elicit targeted immune responses—guided by insights into protective immunity from viral infection—and ultimately support rapid development of more effective vaccines.

#### 3.2.3. Characterizing Immune Dysregulation Underlying Adverse Events (AEs)

The COVID-19 pandemic and the rapid deployment of novel vaccines have prompted a surge of studies investigating vaccine safety. AEs—ranging from mild local symptoms such as pain, swelling, and redness to systemic symptoms like fever and fatigue, as well as severe reactions including anaphylaxis, myocarditis, and autoimmune hepatitis—have been reported following administration of several SARS-CoV-2 vaccines, including the mRNA vaccine BNT162b2, the inactivated vaccines CoronaVac and BBIBP-CorV, and the adenovirus-vectored vaccine AZD1222 [223,224,225,226,227,228]. Here, we use COVID-19 vaccines as a case study to illustrate how single-cell technologies are leveraged to dissect the immune dysregulation underlying vaccine-associated AEs. While clinical trials primarily monitor such events through symptom reporting, single-cell profiling enables the identification of cellular and molecular features that distinguish protective from pathogenic immune responses.

Nineteen-parameter flow cytometry was used to analyze PBMCs from individuals vaccinated with two doses of BNT162b2, quantifying 15 distinct immune cell subsets to investigate early dynamics of innate cells associated with severe systemic symptoms or neutralizing titers [229]. Significant reductions in the DC subsets cDC3 and CD11c^−^Axl^+^Siglec-6^+^ DCs after the second dose correlated with more severe systemic symptoms, whereas decreases in CD16^+^ and CD56^high^ NK cells and non-classical monocytes correlated with higher neutralizing titers. Notably, both patterns were linked to elevated levels of IFNγ and IFNγ-inducible chemokines (MCP-1, IP-10, MIG). Flow analysis using a separate 25-parameter panel showed that AE-associated DC subsets constitutively expressed chemokine receptors CCR2 and CXCR3 prior to vaccination, suggesting they were pre-positioned for rapid recruitment to inflammatory sites upon chemokine induction, thereby contributing to symptom severity. In contrast, NK and monocyte subsets associated with antibody responses only upregulated CCR2 and CXCR3 after vaccination, indicating a vaccine-induced activation linked to enhanced protective humoral immunity. These insights suggest that innate IFNγ-driven chemokine signaling drives both protective immunity and adverse responses through distinct cellular subsets, highlighting a potential targetable pathway for fine-tuning immune cell dynamics to optimize the balance between inducing a protective immune response and minimizing AEs.

In a patient with acute mixed hepatocellular/cholestatic hepatitis after the first dose of BNT162b2 mRNA vaccine and severe autoimmune hepatitis (AIH) after booster, IMC analysis of liver tissue collected 26 days post-boost revealed a dominant infiltration of activated cytotoxic CD8^+^ T cells (GZMB^+^) broadly distributed throughout the liver parenchyma [86]. Although other immune populations—including CD4^+^ T cells, B cells, plasma cells, and myeloid cells—were enriched relative to controls, CD8^+^ T cells were the most abundant, contrasting with typical spontaneous AIH, where B cells and plasma cells predominate. Flow cytometry further showed that intrahepatic spike-specific CD8^+^ T cells were enriched for tissue residency (CXCR6, CD69, CD103) and activation markers (CD38). In the periphery, spike-specific CD8^+^ T cell activation—marked by CD38, GZMB, and T-bet expression—positively correlated with greater clinical severity of hepatitis, and their decline paralleled symptom improvement following immunosuppressive therapy. This study demonstrated that the booster dose triggered severe AIH through activation of spike-specific CD8^+^ T cells, distinguishing it from coincidental or drug-induced liver injury. It underscores the potential risk of disease exacerbation upon re-exposure to the vaccine in individuals who develop hepatic symptoms after the initial dose.

scRNA-seq and scATAC-seq of PBMCs from one individual with acute myocarditis following BNT162b2 vaccination revealed distinct epigenomic and transcriptomic shifts [62,230]. scRNA-seq showed the most pronounced transcriptomic changes in classical and intermediate monocytes, enriched for pathways related to cytokine responses and immune activation. In contrast, scATAC-seq identified the most dynamic chromatin accessibility changes in cytotoxic NK and effector CD8^+^ T cells. A global increase in the activity of RUNX2 and RUNX3 motifs, key regulators of development and differentiation of blood cells, with RUNX3 as the dominant driver, was observed across cell types despite minimal changes in their mRNA expression. Both scRNA-seq and scATAC-seq revealed a consistent downregulation of type I and II IFN signaling—marked by reduced IRF and STAT motif activity and decreased IFN-related gene expression—accompanied by upregulation of pro-inflammatory cytokine programs including IL-1, IL-6, IL-17, and IL-21. This shift from IFN-driven antiviral responses to heightened inflammatory cytokine signaling may reflect a dysregulated immune state during acute myocarditis, potentially contributing to tissue damage through excessive inflammation rather than protective immune activation. Similarly, scRNA-seq was used to analyze two individuals who experienced distinct adverse events during Phase I/II trials of inactivated COVID-19 vaccines: one with fever after receiving BBIBP-CorV and the other with anaphylactic shock after CoronaVac [231]. The fever case showed excessive type I IFN responses and a significant increase in cytotoxic CD8^+^ T cells and MKI67^high^ CD8^+^ T cells. The anaphylactic shock case was characterized by elevated S100A9^high^ monocytes, linked to neutrophil activation and degranulation, as well as increased PPBP^high^ megakaryocytes, suggestive of platelet activation associated with allergic responses.

Together, these studies demonstrate how single-cell technologies have begun to elucidate the immune mechanisms underlying vaccine-associated AEs across platforms and clinical manifestations. The combined insights from these AE-focused studies and analyses of both vaccine- and infection-induced responses and the intrinsic immunogenicity of individual vaccine components (as discussed in previous sections) will enable more targeted design strategies that balance efficacy and tolerability [232]. Moreover, the occurrence of severe AEs in some individuals and the diversity of immune features contributing to these outcomes highlight the need to move toward more stratified vaccination approaches, potentially guided by individual immune profiles.

#### 3.2.4. Evaluating Vaccine Responses in Immunocompromised Populations

While vaccine evaluation in healthy individuals provides a foundation for characterizing immunogenicity (as discussed in Section 3.2.1, Section 3.2.2 and Section 3.2.3), immunocompromised populations—such as older adults, cancer patients, individuals with autoimmune or inflammatory diseases, and transplant recipients—remain underrepresented in clinical trials, despite facing higher risks of severe infection [233,234]. These individuals often exhibit diminished or atypical immune responses due to immunosenescence, immunosuppressive therapies, or chronic immune dysregulation [235,236,237,238,239,240]. As a result, protection cannot be reliably inferred from data in the general population. Single-cell analysis addresses this gap by enabling high-resolution evaluation of vaccine-induced immune responses in immunocompromised populations, uncovering cell-type-specific deficits, dysfunctional activation states, and altered cytokine programs.

CyTOF analysis of PBMCs following the 2016 seasonal influenza A vaccine revealed that patients with myeloproliferative neoplasms had reduced frequencies of central memory CD4^+^ and CD8^+^ T cells, total memory B cells, and resting memory B cells compared to healthy controls (HCs) three weeks post-vaccination [239]. Patients with myeloproliferative neoplasms also exhibited a lower frequency of Tregs but a higher frequency of naïve CD4^+^ T cells, suggesting a delayed or suboptimal immune response. Similarly, spectral flow cytometry analysis of PBMCs from patients with immune-mediated inflammatory diseases receiving methotrexate, an immunosuppressant drug, compared to HCs after vaccination with BNT162b2 showed significantly reduced induction of activated CD8^+^ T cells, contributing to lower vaccine efficacy in the patient group [241]. In another study, 13-parameter flow cytometry analysis of PBMCs revealed that BNT162b2 elicited minimal IFNγ^+^, IL-4^+^ or CD40L^+^IL-21^+^ (circulating Tfh type) CD4^+^ T cell responses in lung transplant recipients [240]. These individuals also exhibited significantly lower frequencies of spike-specific polyfunctional CD4^+^ T cells compared to HCs. scRNA-seq further showed a lack of induction of an interferon-stimulated monocyte cluster seen in HCs, along with attenuated upregulation of gene modules associated with DC activation and antigen presentation. Together, these findings highlight that immunocompromised individuals exhibit distinct cellular and cytokine response deficits following vaccination, underscoring the need for tailored strategies to enhance vaccine efficacy in these vulnerable populations.

While medically induced immunosuppression causes selective, treatment-specific impairments in vaccine immunity, aging involves a gradual, systemic decline in immune function—immunosenescence—that broadly impacts both innate and adaptive responses, contributing to reduced vaccine efficacy across the older adult population [235,242,243]. CyTOF analysis of PBMCs from adults aged 21 to 63, conducted as part of a nationwide post-market surveillance study in Korea (analogous to a Phase IV trial), revealed a weak age-associated decline in B cell subsets, including naïve, memory, plasma cells, plasmablasts, and spike-specific B cells, following two doses of AZD1222 and a BNT162b2 booster [244]. In contrast, naïve CD8^+^ T cells declined significantly with age, while memory T cell subsets—such as memory CD4^+^, CD8^+^ TCM, TEM, TEMRA, and TFH cells—increased, suggesting that differentiated memory T cells may compensate for reduced naïve T cell pools in older individuals. In another study, spectral flow cytometry and scRNA-seq profiling after the same vaccine regiment showed that compared to younger adults (<70), older adults (≥70) had higher frequencies of spike-specific atypical memory B cells, which are characterized by expression of the proteins CD11c and FCRL5 and upregulation of *TBX1* and *ITGAX* transcripts [68]. Furthermore, in older adults, this B cell subset was associated with lower neutralizing antibody titers. Notably, in individuals who received three BNT162b2 mRNA vaccine doses (without the adenoviral AZD1222 priming), such age-related differences in neutralization and B cell subset were not observed, suggesting that vaccine platform and regimen can modulate age-related immune outcomes.

Together, these studies reveal diverse immune deficits across immunocompromised populations, highlighting the need for stratified vaccine strategies. Insights from single-cell technologies can inform clinical decision-making, such as selecting the most appropriate vaccines and optimizing regimens, to improve protection in these vulnerable groups.

### 3.3. Refine Vaccine Manufacturing

Following regulatory approval, a vaccine must be manufactured at high yield and large scale to ensure sufficient supply to meet demand. Viral vaccine production typically requires the utilization of insect or mammalian cell culture [245]. A discussion of the applications and nuances of insect vs. mammalian production cell lines can be found in this review [246]. Several research groups have leveraged insights from bulk analyses to engineer cell lines with improved production capacities. For example, a genome-wide RNA interference (RNAi) screen in a Vero vaccine production cell line identified several host genes that restrict poliovirus production, and silencing these targets resulted in a 20- to 50-fold increase in viral yield [247]. More recently, single-cell approaches have been leveraged to evaluate heterogeneity within production cell lines, offering higher-resolution insights that may guide the engineering of cell lines for optimized vaccine production.

In biologics manufacturing, production cell lines must achieve high yield while maintaining long-term stability, as instability can cause significant variability in protein expression and adversely affect product quality. To evaluate the underlying drivers of cell line instability, a scRNA-seq analysis of a Chinese hamster ovary (CHO) cell line that experienced production instability and significant decrease in monoclonal antibody production identified 9 cell phenotypes within the cell line [71]. Some cells maintained high mRNA expression of both the heavy and light chains, while divergent cells first experienced significantly reduced expression of the heavy chain, followed by reduced light chain expression. Divergent cells revealed a significant decrease in the mRNA expression of gene groups associated with protein synthesis, folding, and stability while the expression of a gene group associated with apoptosis and cellular stress (e.g., *Hmox1, Fth1, Psap*) increased. This information could be used to monitor cell line stability over time and enable early detection of divergent transcriptional programs that compromise protein production.

In a separate study, a scRNA-seq analysis of High Five insect cells infected with recombinant baculovirus to produce influenza VLPs revealed heterogeneous gene expression patterns across cells [78]. The single-cell analysis enabled tracking of different infection states within individual cells based on temporal changes in baculovirus mRNA expression. This approach uncovered significant enrichment of the endocytosis pathway during infection, with early upregulation of clathrin-mediated endocytosis-associated genes, as determined by increased mRNA expression of clathrin *cltc* and actin-related *arpc5* and *capza1*. Additionally, mRNA expression of genes involved in the heat shock response and proteosome pathways, key mediators of protection from proteotoxic stress, was upregulated at early infection stages. The citrate synthase gene (*cs*), essential for aerobic energy production, also showed increased mRNA expression during this period. Given that baculovirus entry relies heavily on clathrin-mediated endocytosis [248], that enhancing the host heat shock response can improve baculovirus productivity [249], and that citrate synthase is critical for baculovirus replication and protein production [250], the genes identified in this scRNA-seq study represent promising targets for overexpression to enhance cell susceptibility to baculovirus infection and improve influenza VLP yield. A similar scRNA-seq study of Sf9 insect cells producing recombinant AAV via dual-baculovirus infection revealed heterogeneity in the heat shock response among infected cells [251]. Infected cells also exhibited upregulation of microtubule mobility genes and downregulation of genes involved in metabolism, ion transport, and apoptosis inhibition, highlighting additional candidate targets for optimizing baculovirus infection and enhancing vaccine production.

## 4. Future Directions

Future directions for single-cell technologies in vaccine research are poised to further enhance our understanding and control of immune responses, vaccine safety and efficacy, and production quality. Advances in single-cell multi-omics that combine transcriptomics, epigenomics, and proteomics analyses will provide a more comprehensive view of cellular states and functions, enabling deeper insights into the mechanisms of vaccine-induced immunity. Furthermore, single-cell analyses hold great promise for guiding the development of personalized vaccines by identifying individual-specific immune profiles and tailoring vaccine design or selection accordingly. As single-cell technologies continue to expand our understanding of the unique immune responses generated by various adjuvants, epitopes, RoAs, and vaccine platforms, we can begin to develop a comprehensive vaccine design “toolbox” that streamlines the development of tailored vaccines based on the unique characteristics of the target virus. This will support the rapid development of vaccines that elicit robust protection and would be particularly promising for at-risk patient groups such as the elderly, obese, and immunocompromised. On the manufacturing side, single-cell approaches have the potential to support the real-time monitoring and engineering of production cell lines, improving yield, quality, and reproducibility.

Given the large data sets provided by single-cell approaches, improving the scalability and throughput of single-cell technologies and developing novel bioinformatic and machine learning tools for data interpretation will be essential for translating these insights into actionable outcomes in both clinical and industrial settings. Additionally, artificial intelligence (AI) and machine learning (ML) are poised to significantly enhance the use of single-cell technologies by enabling more precise, efficient, and comprehensive analysis of large-scale, complex datasets. These technologies can significantly streamline data analysis, enable the identification of novel cellular subsets and biologically significant trends, and support vaccine design and evaluation. By integrating multi-omic data with systems immunology approaches, researchers can build predictive models that forecast vaccine outcomes and optimize vaccination strategies for a wide range of subpopulations, ultimately advancing precision vaccinology. As these single-cell technologies and data analysis approaches continue to improve, they are expected to play a central role in the design and refinement of next-generation vaccines.

## 5. Conclusions

Single-cell technologies have emerged as powerful tools across multiple stages of the vaccine development pipeline. By enabling high-resolution transcriptomic, proteomic, and epigenomic analysis at the single-cell level, these approaches provide valuable insights into viral tropism, immunogenic viral epitopes, protective immune signatures, and heterogeneity across human subpopulations. Such information can directly inform rational vaccine design. Additionally, single-cell analyses provide critical insights into vaccine performance and correlates of protection, variability across subpopulations, and rare cellular responses that bulk methods may overlook. In the context of vaccine manufacturing, single-cell technologies facilitate the in-depth characterization of production cell lines, with the potential to guide the optimization of cell line stability and productivity. Collectively, these applications highlight the emerging utilization of single-cell technologies to accelerate the development, evaluation, and manufacturing of next-generation vaccines.

## Figures and Tables

**Table 1:** Common single-cell technologies in infection and vaccination studies.

	Technology	Insights	Example Applications	Throughput and Multiplexity	Review Papers
Epigenomic	scATAC-seq	Chromatin accessibility	Identify chromatin alterations induced by infection and vaccination [62] Characterize regulatory elements controlling immune responses [62] Discover epigenetically distinct immune cell types and novel subsets [63]	~10^2^–~10^5^ cells/experiment [64] ~2500–73,000 reads/cell [65]	[66,67]
Transcriptomic	scRNA-seq	Transcript diversity	Identify immune cell subsets and infer effector states, detect rare cellular subsets [68] Identify infection or vaccine induced transcript expression signatures [69] Simultaneously detect host and pathogen RNA [70] Characterize cell line heterogeneity [71]	~10^2^–~10^5^ cells/experiment [72] 5–30% transcriptome coverage [73] 1000–14,000 genes/cell [74,75] 1–3 days/experiment [74]	[76,77]
	scVDJ-seq (scBCR-seq and scTCR-seq)	B/T-cell receptor clonotype diversity	Characterize B/T-cell receptor clonotypes heterogeneity, specific to infection or vaccination [78] Identify antigen-specific dominant B/T-cell receptor sequences associated with protection and deduce immunodominant antigens [79] Track B/T-cell differentiation and trace lineage (combined with scRNA-seq) [69]		[80,81,82]
Proteomic	Flow cytometry	Fluorescence-based protein expression measurement in suspended cells	Identify immune cell subsets and functional states Measure proliferation, detect cytokine and antibody production [83] Detect infected cells, intracellular pathogens, and apoptosis [84] Flow cytometry: Characterize protein expression levels of cell lines [85] IMC: Track immune cells interaction, infiltration and tissue remodeling [86]	~10^5^ cells/s [87] Conventional: 5–10 markers, up to 28 [88,89] Spectral: 20–30 markers, up to 40 [90,91]	[90,92]
	CyTOF	Metal-tag-based protein expression measurement in suspended cells		~1000–2000 cells/s [93] 30–40+ markers [94,95]	[96,97]
	IMC	Metal-tag-based protein expression measurement and localization in tissue		1–5 µm/pixel [96] 20–200 Hz [97] 30–40+ markers [41,98,99]	[40,97]
Transcriptomic + Proteomic	CITE-seq	Integrated transcript diversity and surface protein expression	Identify immune cell subsets and functional states with enhanced cell type resolution Detect activation markers and cytokine production [100] Detect immune dysregulation (e.g., hyperactivation or suppression)	~10^3^ cells/experiment [42] ~10^2^ proteins + transcriptomic data [42]	[101]
